# Bilateral Simultaneous Total Knee Arthroplasty in Elderly Patients with Severe Osteoarthritis of Knee Joint in a Tertiary Care Hospital: A Descriptive Cross-sectional Study

**DOI:** 10.31729/jnma.5742

**Published:** 2021-05-31

**Authors:** Kapil Mani KC, Dirgha Raj RC, Suman Babu Marahatta, Ankit Niroula

**Affiliations:** 1 Department of Orthopedics, Civil Service Hospital, Minbhawan, Kathmandu, Nepal

**Keywords:** *complications*, *orthopedic*, *total knee arthroplasty*

## Abstract

**Introduction:**

Total knee arthroplasty is one of the most successful orthopedic surgeries performed in recent decades. However, there are controversies regarding the simultaneous or staged bilateral total knee arthroplasty. The aim of this study is to find the prevalence of bilateral total knee arthroplasty in elderly patients among severe osteoarthritis of knee joints in a tertiary care hospital.

**Methods:**

This is a descriptive cross-sectional study conducted from hospital records of 2015 to 2019 in elderly patients with severe osteoarthritis in a Tertiary Care Hospital. Ethical clearance (20/2020) was taken from Institutional Review Board. Convenience sampling was used and statistical analyses were performed using the Statistical Package for the Social Sciences software (version 16.0). Point estimate at 95% confidence interval was calculated along with frequency and proportion for binary data.

**Results:**

Out of 1200 patients with severe osteoarthritis, the prevalence of bilateral total knee arthroplasty was found to be 80 (6.67%) (95% Confidence Interval = 6.60-6.74). The mean Knee Society Score was 36 ±3.70 preoperatively. There were 21 (26.2%) patients having hypertension, 17 (21.2%) diabetes mellitus, 14 (17.5%) chronic obstructive pulmonary disease and 7 (8.7%) coronary artery disease.

**Conclusions:**

Bilateral simultaneous total knee arthroplasty was required in less patients with severe osteoarthritis of knee joints. Bilateral simultaneous total knee arthroplasty is safe, convenient, effective with early functional recovery, higher patient satisfaction and cost effective with acceptable cardiac, pulmonary and neurological complications in properly selected patients.

## INTRODUCTION

Total knee arthroplasty (TKA) significantly reduce the pain, restore the physical activities and improve the qualities of life in patients with end stage arthritis of knee joint.^1,2^ Bilateral TKA can be performed either simultaneously under the same anesthetic set up or as a staged procedure under separate anesthetic set up at different time interval and even at different hospital.

Bilateral simultaneous total knee arthroplasty (BSTKA) has been found as convenient and safe technique with early functional recovery, higher patient satisfaction rate and significantly cost effective.^3-5^ However, other studies have mentioned the increased cardiovascular problems, neurological complications, wound breakdown, deep infection, increased intra-operative blood loss, greater need of blood transfusion, and increased mortality associated with BSTKA.^3,6-9^ Even though staged bilateral TKA may decrease the potential complications, it causes higher hospitalization costs.^3,9^

The aim of this study is to find the prevalence of bilateral total knee arthroplasty in elderly patients among severe osteoarthritis of knee joints in a tertiary care hospital.

## METHODS

This was a descriptive cross-sectional study based on hospital record in Civil Service Hospital from 2015 to 2019. Data was collected form hospital record (2015 to 2019) after getting permission from the institutional review board of our hospital with ethical clearance number 20/2020. Convenience sampling was used and the sample size was calculated as,


$$\begin{array}{ll} \text{n} & \text{= }{\text{Z}}^{\text{2}}\times \text{p}\times q\;/{\text{e}}^{\text{2}}\\ & \text{= }{\left(1.96\right)}^{\text{2}}\times (0.5)\times (1-0.5)/{(\text{0.04)}}^{\text{2}}\\ & \text{= }600 \end{array}$$

Where,

n = minimum required sample size
Z = 1.96 at 95% Confidence Interval (CI)
p = prevalence taken as 50% for maximum sample size
q = 1-p
e = margin of error, 4%

Since convenience sampling was used, sample size was doubled. Therefore 1200 patients were enrolled in our study.

Those patients treated with stage bilateral or unilateral TKA as well as revision procedures were excluded from the study. Those with more than 60 years and less than 80 years of age were included in the study. Associated comorbidities like hypertension, diabetes mellitus, coronary artery disease (CAD), chronic obstructive pulmonary disease (COPD), liver disease, kidney disease, thyroid diseases and rheumatoid arthritis were recorded during preoperative checkup. In addition to this patient's age, sex, height, weight, body mass index (BMI), Knee Society Score (KSS), American Society of Anesthesiologist (ASA) grade, date of surgery, tourniquet time, peri and post-operative complications, pre and postoperative hemoglobin, amount of blood loss within 48 hours in drain, amount of blood transfusion were noted.

Statistical analyses were performed using the Statistical Package for Social Sciences software (version16.0). Quantitative variables were documented as mean±standard deviation.

## RESULTS

Out of 1200, the prevalence of bilateral total knee arthroplasty was found to be 80 (6.67%) (95% Confidence Interval = 6.60-6.74). The mean Knee Society Score (KSS) was 36±3.70 preoperatively. The average age of the patients was 67.64±4.13 and BMI was 25.71±2.04. Mean blood loss in both knees was 820.56 ± 112.27ml, average hospital stay was 8.16±1.82 day. The demographic profiles, ASA grading and associated comorbidities were demonstrated in (Table 1).

**Table 1:** Showing demographic profiles, ASA grading and associated comorbidities.

Parameters	n (%)
Sex	
Male	29 (36.3)
Female	51 (63.7)
ASA	
Grade I	23 (28.8)
Grade II	38 (47.5)
Grade III	19 (23.7)
Associated Comorbidities	
HTN	21 (26.2)
COPD	14 (17.5)
CAD	7 (8.7)
DM	17 (21.2)
Hypothyroidism	9 (11.2)

Intra-operative blood loss, tourniquet time, pre-operative and post-operative hemoglobin and hospital stay (Table 2).

**Table 2:** Showing intra-operative parameters and hospital stay.

Parameters	Mean±SD
Tourniquet time in more severe side (min)	65.2±3.92
Tourniquet time in less severe side (min)	58.4±3.35
Total blood loss in both knees (ml)	820,56±112.27
Pre-operative Hb (gm/dl)	13.73±0.75
Post-operative Hb (gm/dl)	9.23±0.41
Hospital stay (days)	8.16±1.82
Patients with blood transfusion	46 (57.5)

Similarly post-operative complications were mentioned (Table 3).

**Table 3:** Showing the post-operative complications.

Postoperative complications	n (%)
POCD	22 (27.5)
Chest infection	12 (15)
Intra-operative fracture	1 (1.2)
Renal dysfunction	8 (10)
Superficial infection of wound	7 (8.7)
Deep infection of wound	1 (1.2)
Urinary tract infection	7 (8.7)
Deep vein thrombosis	1 (1.2)
Pulmonary embolism	0 (0)
Myocardial infarction	0 (0)
Myocardial ischemia	5 (6.2)
Mortality	0 (0)

## DISCUSSION

In the present context, TKA has been considered gold standard treatment for end stage arthritis of knee joint.^10,11^ Whether to perform the simultaneous or staged TKA is a matter of controversy and debated for many years. Some centers have preferred the simultaneous procedures while others have questioned the indications for this procedures.^12-14^ With development of recent advances and techniques, peri and post-operative complications have been found below acceptable limits.^15^ In our study many patients had been associated with comorbid conditions like hypertension 26.2%, diabetes mellitus 21.2%, COPD 17.5% and CAD 8.7%. Even though some of these patients had more than one major disease, we did not have major complications related to the surgery, however we performed thorough preoperative assessment and perioperative monitoring in all these cases.

In our study mean age of the patients was 67.64±4.13 years. Cahill, et al^16^ demonstrated that mean age of the patients in their studies was 83.5 years while mean age of the patients in the study of Mnatzaganian, et al^17^ was 76.3±4.5 years. Since the life expectancy of Nepalese population is lowered as compared to the western population this mean age in our study looks significantly older and comparable to other studies. ASA grade is one of the most useful indicators regarding the patient's health status and it is highly correlated to the postoperative complications and even mortality of patients.^18^ Higher the preoperative ASA grade, more the postoperative complications. In our study 76.3 % of patients were ASA grade I or II. This is why the mortality and morbidity in our study are relatively low.

Mortality in our study was nil. Results of Liu L, et al.^1^ revealed a combined mortality rate of 0.32% for simultaneous bilateral TKA (0.37%) and staged bilateral TKA (0.28%). Some studies have shown increased mortality rate for simultaneous TKA compared with staged TKA.^19^ A number of more recent studies have shown no difference in mortality between simultaneous and staged TKA which may correlate with the improvement in surgical technique over time or better patient selection, although some had relatively small numbers.^20^ Similarly pulmonary embolism (PE) was zero and there was a single case of DVT in our study. However It has been found that pulmonary embolism is dominant cause of mortality in simultaneous TKA than staged because of increased embolic load in simultaneous surgery. The use of pneumatic tourniquet, intramedullary devices and cement during surgery further enhance the embolic phenomenon. Larson, et al.^21^ showed the PE is nil in their study undergoing the simultaneous TKA treated with 60mg of Aspirin twice daily and thigh foot intermittent compression devices. Meanwhile prophylaxis has become standard care for reducing the deep vein thrombosis (DVT), PE and even mortality which can be usually achieved with combination of mechanical device and chemotherapeutic agent.^22,23^

Regarding the cardiac complications, there were no cases of congestive cardiac failure and myocardial infarction, but 5 (6.2%) cases of myocardial ischemia in our study. Cardiac complications like congestive cardiac failure, myocardial infarction and arrhythmias are some of the common problems after BSTKA.^24^ The causes for cardiac problems remain unclear however pre-existing comorbid diseases and elderly patients more than 80 years are more at risk. It is sometimes assumed that increased physiological stress imposed by simultaneous procedure in high risk patients with compromised cardiorespiratory reserve could be the possible cause for the cardiac complications.^25^

Number of patients with post-operative confusion disorder (POCD) in our study was 22 (27.5%) which is the commonest problem reported in our study. A higher rate of postoperative neurological complications in the simultaneous bilateral group could be partly explained by a number of factors, including increased postoperative blood loss, increased hypoxemia and anemia, increased need for analgesics, and increased luid shifts and potential electrolyte imbalances. Several authors have shown that bilateral procedures result in an increased prevalence of fat emboli with resulting neurological and pulmonary effects.^26^

Forty-six (57.5%) patients in our study required blood transfusion for BSTKA. Blood transfusion after simultaneous surgery is often required and the decision to transfuse blood depends upon pre and post-operative hemoglobin, amount of blood loss in drain, as well as clinical assessment of patients and predetermined threshold. Belmar, et al.^27^ reported blood transfusion in all the 15 patients in his study of unilateral TKA while Petruccelli, et al.^28^ mentioned 67% of his patients required blood transfusion in primary unilateral TKA. Perioperative use of tranexamic acid and local infiltrative analgesia (LIA), release of tourniquet with adequate cauterization of the bleeders before closure of skin are definitively helpful to decrease the postoperative blood loss. The relatively low amount of blood transfusion requirement in our study is because of use of tranexamic acid locally in all cases. There was a case of intra-operative fracture of medial condyle which was managed by internal fixation with satisfactory outcomes. Similarly quite significant number of patients developed chest infection (15%) and urinary tract infection (8.7%) within 7 days of post-operative period which were accordingly managed with culture sensitivity and appropriate antibiotics. Renal dysfunction was found in 10% of patients which was temporary in all cases and managed by physician. Superficial infection was noted in 8.7% of patients that were treated by suitable antibiotics and dressing except in one cases who required debridement. Deep infection was developed in one case (1.2%) which was treated two stage revision procedure.

The study of Petruccelli, et al.^28^ showed that KSS was improved from (42.3±17.8) to (86.45±3.82) within one year interval. On the basis of KSS the results of this study were similar to other previously published studies in terms of improvement of quality of life and functions, relief of pain and restoration of patient's independent movement.

## CONCLUSIONS

Bilateral simultaneous total knee arthroplasty was required in less patients with severe osteoarthritis of knee joints. It is safe, convenient, effective with early functional recovery, higher patient satisfaction and cost effective with acceptable cardiac, pulmonary and neurological complications in properly selected patients. Both patients and family members should be properly informed regarding merits and demerits of the surgery. However we do not recommend the simultaneous knee arthroplasty in every patients over the staged procedure because there are risks and benefits of each of these techniques and these potential problems should be analyzed in light of each individual needs and concern.
